# CGAP-Align: A High Performance DNA Short Read Alignment Tool

**DOI:** 10.1371/journal.pone.0061033

**Published:** 2013-04-11

**Authors:** Yaoliang Chen, Ji Hong, Wanyun Cui, Jacques Zaneveld, Wei Wang, Richard Gibbs, Yanghua Xiao, Rui Chen

**Affiliations:** 1 School of Computer Science, Fudan University, Shanghai, China; 2 Human Genome Sequencing Center, Department of Molecular and Human Genetics, Baylor College of Medicine, Houston, Texas, United States of America; Indiana University, United States of America

## Abstract

**Background:**

Next generation sequencing platforms have greatly reduced sequencing costs, leading to the production of unprecedented amounts of sequence data. BWA is one of the most popular alignment tools due to its relatively high accuracy. However, mapping reads using BWA is still the most time consuming step in sequence analysis. Increasing mapping efficiency would allow the community to better cope with ever expanding volumes of sequence data.

**Results:**

We designed a new program, CGAP-align, that achieves a performance improvement over BWA without sacrificing recall or precision. This is accomplished through the use of Suffix Tarray, a novel data structure combining elements of Suffix Array and Suffix Tree. We also utilize a tighter lower bound estimation for the number of mismatches in a read, allowing for more effective pruning during inexact mapping. Evaluation of both simulated and real data suggests that CGAP-align consistently outperforms the current version of BWA and can achieve over twice its speed under certain conditions, all while obtaining nearly identical results.

**Conclusion:**

CGAP-align is a new time efficient read alignment tool that extends and improves BWA. The increase in alignment speed will be of critical assistance to all sequence-based research and medicine. CGAP-align is freely available to the academic community at http://sourceforge.net/p/cgap-align under the GNU General Public License (GPL).

## Introduction

In recent years, advances in sequencing have led to the production of unprecedented amount of sequence data. Alignment, which maps reads to the reference sequence, is one of the most computationally demanding tasks performed in typical sequence data processing. Accurate sequence alignment is critical for SNP calling [1], structural variation detection [2] and further downstream analysis.

In order to efficiently and accurately map large numbers of short reads many new alignment programs have been developed. The algorithms underlying most of these tools can be classified into two major categories [3]. The first category uses hash tables to hash either read sequences, as in MAQ [1], SeqMap [4] and CloudBurst [5], or the genome reference, as in SOAPv1 [6], PASS [7], MOM [8] and ProbeMatch [9]. Although this technique can be easily parallelized, the major drawback of using hash tables is that either they must scan the whole genome, even when few reads are aligned, or they require a large amount of memory to build an index for the reference. The second category is based on string matching using a representation of prefix/suffix trie [2], including suffix tree, suffix array [10], enhanced suffix array [11] and FM-index [12]. The first three representations are used by TRELLIS [13], MUMmer [14] and Vmatch (http://www.vmatch.de). Unfortunately, these programs have poor performance on large-scale references including the human genome due to memory constraints. The FM-index is a type of substring index based on the Burrows-Wheeler transform, with some similarities to suffix array. Owing to its small memory requirement, it is utilized by numerous state of the art programs including SOAPv2 [15], Bowtie [16], Bowtie2 [17] and BWA [18].

BWA has become one of the most widely used alignment tools owing to its efficiency and accuracy. BWA possesses higher recall and precision than SOAPv2 or Bowtie. However, both Bowtie and SOAP2 are significantly faster than BWA. Therefore, it is highly desirable to improve the speed of BWA while maintaining its alignment quality. In this paper, we describe a new efficient alignment tool, CGAP-align, following the framework of BWA. As part of short read mapping in BWA, possible mismatches and gaps are enumerated during the traversal of the FM-index of the reference sequence. The time efficiency of BWA is mainly impacted by two factors. The first is the efficiency of locating a substring in the reference. The second is the ability of the program to bypass segments of the reference where the read would contain a large number of mismatches, avoiding the need to enumerate all possible sets of mismatches and gaps, through a process called pruning. Improvements to these strategies have been implemented in CGAP-align to optimize the efficiency of both the reference querying and the pruning steps.

In this report, we first introduce a novel data structure, Suffix Tarray, which speeds up the process of locating a read on the reference. Second, we present an effective pruning strategy that more accurately predicts the number of mismatches in a read prior to alignment. Pruning is significantly improved by using a set of training reads in advance to identify and study frequent patterns of nucleotides. The performance of CGAP-align was evaluated on both simulated data and several sets of real paired-end sequence data. Our results indicate that by implementing both of these improvements alignment speed is significantly increased without sacrificing recall or precision.

## Methods

### 1.1 Suffix Tarray (STA): Improving Reference Queries

To improve the alignment speed, we utilized a data structure, Suffix Tarray (STA), to index reference sequences in CGAP-align. STA uses a new index structure that is a hybrid of trie (inspired by suffix tree) and suffix array data structures. Before we present the concept of STA, we first briefly review the two most widely-used data structures for sequence indexing: suffix array (SA) and suffix tree (ST).

#### 1.1.1 Background: Suffix Tree (ST) & Suffix Array (SA)

Suffix tree is a data structure that encodes all of the suffixes of a given string in the form of tree, allowing quick location of substrings in O(|*W*|+N) time, where |*W*| is the length of the substring and N is the number of occurrences of that substring. More specifically, ST for a string *X* is a tree whose edges are labeled with strings, such that each suffix of *X* corresponds to exactly one path from the tree's root to a leaf. In the case of sequence data, a suffix tree for string *X* of n characters is queried and constructed in linear time [19]. Owing to its query efficiency, ST was once the predominant data structure for read alignment [2]. However, ST usually requires O(*n*) memory with a large constants leading to a large memory requirement. State-of-art ST methods like TDD [20] and TRELLIS [13] cannot currently scale up to the entire human genome without a disk-based strategy that results in a suffix tree consuming tens of gigabytes of space.

Suffix array (SA) is an array of integers each of which gives the start positions of suffixes of a string *X* in alphabetical order. The alphabetic ordering of SA enables each substring of *X* to be queried through a binary search on SA. Given a string *W* is a substring of *X*, we define the interval [*k, l*] as the SA interval of *W*. In particular, if *W* is empty, the corresponding SA interval is [1, *n*-1], where *n* is the length of *X*. The set of positions of all occurrences of *W* in *X* is thus {SA[*i*] : *k*≤*i*≤*l* }, where SA[*i*] maps the SA position *i* to a reference coordinate. A SA, if optimally implemented, consumes O(*n*) space with a small constant and can be constructed in linear time [21](Ko et al., 2003). Further improving the space efficiency, FM-index [12](Ferragina et al., 2000), a compressed representation of SA, was developed. In FM-index, SA intervals are retrieved by the summing corresponding *C* and *Occ* values based on Burrows-Wheeler transform (BWT) [22] (Burrows et al. 1994). Given a substring *W* and a character *c*, Ferragina and Manzini [12](2000) shows that
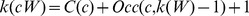


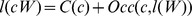
and that *k*(*cW*)≤*l*(*cW*) iff *cW* is a substring of *X*, where [*k*(*W*), *l*(*W*)] and [*k*(*cW*), *l*(*cW*)] are the SA intervals of the substrings *W* and *cW* respectively. During each query, a backward search is performed on FM-index so that the substring *W* is scanned from the end to the beginning. FM-index is constructed in O(*n*) time from the reference *X* (as it can be constructed in linear time from a SA) and used to query a substringfig of length *m* in O(*m*) time. Due to its space efficiency, FM-index has recently been adopted by many widely-used mapping tools, including SOAPv2 [15], Bowtie [16] and BWA [18]. However, such space efficiency comes at the cost of reduced query performance. Empirically, ST is much faster than FM-index for queries on substrings [23].

#### 1.1.2 Overview of the Suffix Tarray (STA) Structure

In an FM-index, for a character *c*, *Occ* is a function of the SA position *y*, which counts the number of the occurrences of *c* within the suffix interval [1, *y*] of the SA. To accelerate the calculation of *Occ* values, the SA is divided into buckets with a fixed size. For the SA positions *y* that define the left bucket boundaries, the corresponding *Occ* values are pre-computed and stored in a table. This allows all other *Occ* values to be efficiently calculated by counting the occurrences of the character *c* from the start of their corresponding buckets instead of from the beginning of the SA.

**Figure 1:**
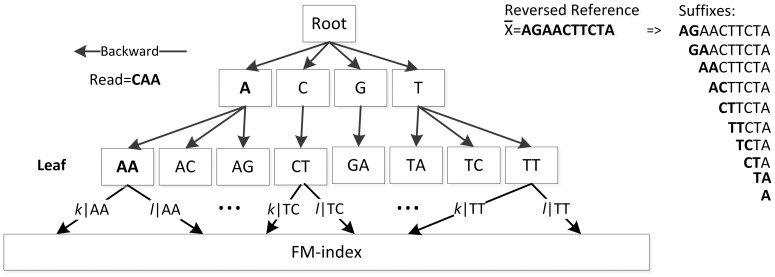
An example Suffix Tarray for the reference sequence “ATCTTCAAGA”. A trie (truncated suffix tree) for “AGAACTTCTA” is built on the top of an FM-index. Each node of the trie stores *k* and *l*, where [*k*, *l*] is the SA interval of the substring that corresponds to the leaf node on the FM-index.

However, retrieving a pre-computed *Occ* value is still much faster than retrieving an *Occ* value in the middle of a bucket. This observation implies that pre-computing frequent Occ values has the potential to speed up the mapping process. The basic idea of Suffix Tarray (STA) is to first find those most frequently visited Occ values (and their C values) and then organize them in a ST so that they can be efficiently accessed during alignment. With this novel approach, we can take the advantage of both the time efficiency of the ST and the space efficiency of FM-index. At a high-level, STA can be viewed as a truncated suffix tree (TST) encoding corresponding SA intervals of a FM-index at each leaf (Figure 1). As the height of the tree is bounded to a reasonably small value, we adopt trie as a light implementation of the TST.

There are two major steps to construct a STA for a reference string X. In the first step, a FM-index is built for the entire reference sequence to support the SA queries from the suffix tree [18]. In the second step, we construct a truncated ST based on the FM-index. All possible suffix strings of the reversed reference sequence x¯ are enumerated. Instead of building the whole ST for x¯, we truncate the ST according to the frequency values of the nodes. The frequency value of a node in ST for x¯ is defined as the number of occurrences of the substring which it represents in x¯. In our example, the frequency value of the node “CT” is 2 since there are 2 prefixes valued “CT” among all of the suffixes of x¯. The nodes in tries will be discarded if their frequency values are smaller than a threshold ε which is calculated by St. We select a maximal ε such that the resulting trie has a size less than a user specified trie size St. This is accomplished by a binary-search style enumeration of the possible values of ε. Initially, the possible range of ε is set to [1, |X|]. The program first tries to construct a trie using a value P in the middle of possible range, such that in the first iteration P is approximately |X|/2. If, during construction, the trie size exceeds the size limit St, then the maximum possible value for ε is reduced to P-1. If, on the other hand, the trie is constructed without exceeding St then the minimum possible value of ε is increased to P. This process is iterated until the possible range of ε is reduced to a single value, which we assign to ε. At last, a truncated trie with frequency threshold ε and size less than St is built. Figure 1 is an example of a STA.

In the current version of CGAP-align, each trie node takes 36 bytes. One concern when building a STA is how to choose an appropriate truncated trie size. If the size is too small, then the index will not see a significant increase in query speed since the FM-index will dominate the query process. Otherwise, memory consumption will increase. Fortunately, since the frequency values of novel nodes decreases rapidly with increasing trie size, a STA with a moderate-sized trie can perform nearly as well as a STA with a large trie. We denote ST_100_ as a full ST for truncated at depth 100 and the frequency ratio of a trie as the values of all nodes in that trie divided by the sum of frequency value of all nodes in ST_100_. According to our experiment, when indexing the human reference genome, a 300 MB sized truncated ST has a frequency ratio of 13.32% while 1 GB has 14.68%.

#### 1.1.3 Matching Substrings to Suffix Tarray



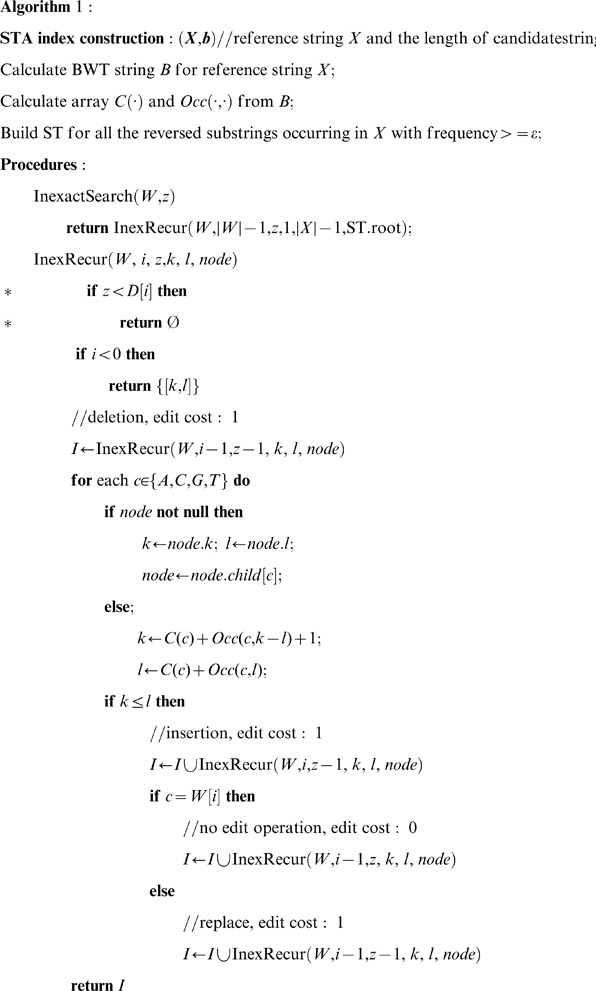



The procedure to query a substring from STA is given in Algorithm 1. Procedure InexactSearch(*W*, *z*) takes substring *W* and maximal edit distance z as input, and returns the SA intervals of substrings that match *W* with no more than z differences. The algorithm framework is similar to that used in BWA, but with a different index structure. The *l* and *k* values are re-calculated based on STA. For a given node of ST, we denote node.*child* as its child-list, while node.*k* and node.*l* define its SA interval values. The lines with an asterisk mark the D-array pruning strategy used in BWA, which will be discussed in section 1.2.

The actual implementation of CGAP-align differs slightly from the pseudo code given in Algorithm 1. CGAP-align inherits the logic of BWA's inexact mapping, where “gap extensions” do not add to the number of edits that have been performed (i.e. IndexRecur will be recursively called with maximum edit distance value z). This helps to detect relatively long gaps. However, it also causes a potential issue resulting in false negative alignments in both BWA and CGAP-align. This problem is addressed in section 2.2.

### 1.2 Data-Conscious D-Array Calculation (DCDC): Improving Pruning

**Figure 2:**
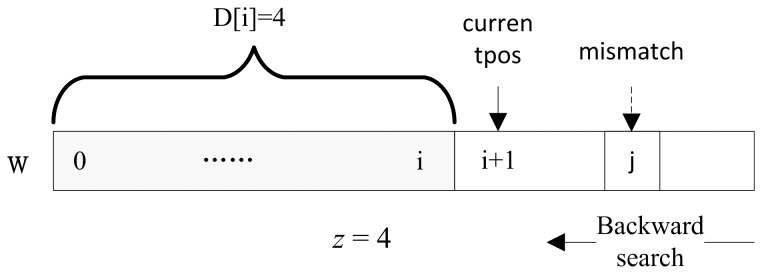
An example of D-Array based pruning. The maximum allowed number of differences, *z*, is 4. When backward search reaches position *i*+1, the algorithm detects that at least 4 differences must exist (as indicated in D[*i*]), while only three are allowed according to *z* due to the previous mismatch at position *j*.

To improve the efficiency of mapping, a pruning step is implemented in BWA. A lower bound is calculated for the number of differences between *W*[0,*i*] (i.e. the *i*-length prefix of the read *W*) and any substrings of the reference sequence and stored as the *i* ^th^ element of array D, all prior to mapping. Reads with D[i] greater than the maximum tolerated number of mismatches defined by the user are excluded from further alignment. If calculating D-array is faster than the time it would take to map the pruned part of the search trees, pruning improves mapping efficiency. The degree of improvement depends on both the speed at which the D-array can be calculated and the accuracy of the estimation of D-array. A more accurate estimation of D results in a smaller search space and more efficient mapping. Figure 2 gives an example of how D-array would work during a backward search on either FM-index or STA. D-array prunes superfluous enumerations before mapping, providing a significant boost in performance.

#### 1.2.1 D-array: Background

BWA proposes a method to estimate the D-array by splitting W into several small strings. Let e(*W*) be the minimal number of the edit operations required to make *W* exactly align onto the reference *X*. BWA divides *W* into segments *w*_1_ *w*_2_…*w*_t_, where e(*w_p_*) = 1 for 1≤*p*<*t* and e(*w_p_*)≤1 for *p* = *t*. Then D[*i*] is approximated as *p*-1 for 1≤*i*<|*W*|, where *w_p_* contains the (*i+1*)^th^ element of *W*, or *t* for *i* = |*W*|. For example, given a reference *X* = “AACGTATCGACG” and a read *W* = “AACTGA”, BWA segments the *W* as “(AACT)(GA)” and thus produces the D-array “000111”. The time to calculate the D-array for read W is in O(|*W*|) when the FM-index of the reverse of X is used. In CGAP-align, the calculation of D-array is further accelerated by using STA.

#### 1.2.2 A Tighter Lower Bound for D-array

Identifying a *w_p_* with e(*w_p_*)>1 when splitting *W* generates a D-array with a tighter lower bound. In the example described above, if we consider segments with an e(*w_p_*) equal to 2, then we derive the segmentation “(AACTG)(A)” with the corresponding D-array “000122”. By allowing segments with more mismatches we derive a tighter bound for D.

However, the above tighter bound comes at the cost of longer computations. The time cost of verifying e(*W*) = *k* is exponential to the value of *k*. Therefore, it is too expensive to calculate each e(*w*_i_) value on the fly unless e(*w_i_*) is strictly restricted to be 1 (in this case calculation of e(*w*) is O(|*w*|)). To address this issue, we add a pre-processing step, in which we identify the frequent substrings from the training read set with a e(*w*) = 2. This approach is feasible as genomic sequences from the same species are similar among individuals. Thus, by providing a training read set from each species, it is possible to generate a list of species specific frequently occurred substrings (*w*) with e(*w*) = 2.

In a pre-processing phase, an appropriate number of patterns with the greatest frequency values are identified by a depth-first visit on an FM-index built upon the training reads and then organized into an Aho-Corasick (AC) automaton [24]. The number of patterns is determined by the AC automaton size specified by the user. Given a read *W*, an AC automaton helps to find all substrings of *W* (denoted as *w*) that match to any of the indexed patterns (denote as *f*) in linear time. A description of AC automaton is given in section 1.2.3. For the sake of the efficiency, we only index patterns with e(*f*) = 2 into AC automatons.

#### 1.2.3 Aho-Corasick Automaton

**Figure 3:**
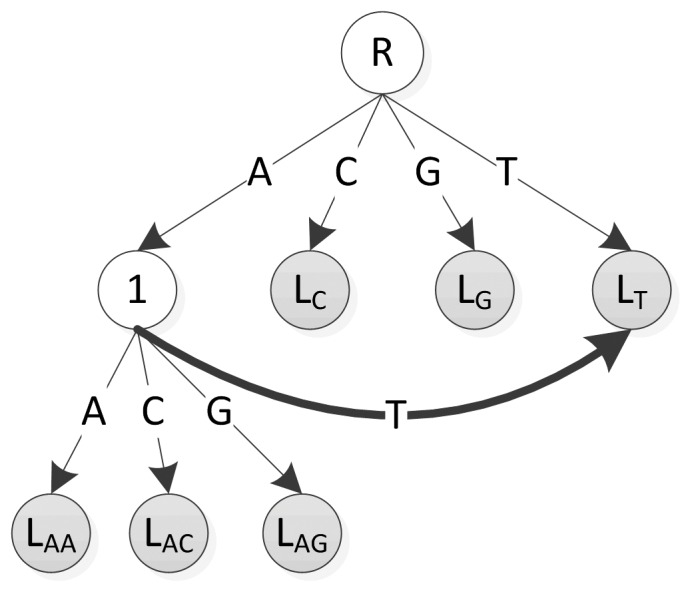
An example AC automaton. The frequent pattern string set *F* is {AA, AC, AG, C, G, T}. The grey nodes represent the leaf states. The bold “T” edge is connected as among all the prefixes of the 6 patterns, “T” is the longest suffix of “AT”. Given a short read *W* = “CAT”, the state transition sequence when querying W from the automaton is <R, L_C_, R, 1, L_T_>, which indicates the occurrence of frequent patterns ‘C’ and ‘T’.

In general, millions of frequent patterns (FP) with e(*f*) = 2 are found in the pre-processing phase. We use a AC automaton G_T_ to organize these FPs so that when given a read *W*, we are able to quickly query all of the FPs that are contained in *W* in a single scan. This allows for efficient segmentation of *W* according to the FPs it contains. We adopt the AC algorithm [24] in which the FPs are organized in an AC automaton. Instead of searching for occurrences of a single string *f* within a main text string *W*, AC automaton supports searching for occurrences of a set of strings *F* within *W*. An AC automaton makes use of the information embodied by the string set *F* itself to determine where to begin then next matching attempt if a mismatch happens, thus bypassing re-examination of previously matched characters in *W*. There is a particular type of state called a “leaf state”, each of which corresponds to a string *f*. The transition to a leaf state L_f_ indicates an occurrence of *f* in *W*. When a match is detected, we reset the automaton to its initial state and continue the scanning on *W*. These steps are iteratively performed until we meet the end of *W*. A simple example is shown in Figure 3.

#### 1.2.4 Calculating the Better D-array



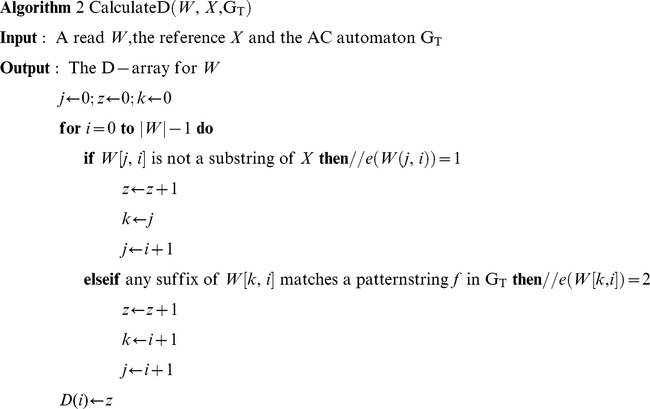



Algorithm 2 shows the detailed procedure to estimate a tighter D-array in CGAP-align. We use a greedy strategy to iteratively find the first matched pattern string f of *W* from the AC automaton G_T_. If no such *f* is found, we segment W by e(*W*[j, i]) = 1 instead (the first “if” condition). As described above, when we find a match in G_T_ at position i, we reset the automaton, scanning matches within *W*[i+1, |*W*|-1] in the following comparison.

## Results

As described above, two major changes have been added to the current version of BWA. First, instead of FM-index we utilize STA, a novel data structure that speeds up string matching by constructing a trie on top of an FM-index. Second, we implement a new pruning method DCDC. As mapping time increases exponentially with the number of mismatches, it is highly desirable to prune reads with large numbers of mismatches. To achieve this goal, a data training process has been implemented to identify a set of frequent substrings with two mismatches from the genome. This pre-processed data allows for accurate calculation of the minimal number of mismatches between a prefix and the genome, resulting in further reduction of the search space.

Both functionalities have been implemented in C. To facilitate the usage of CGAP-align, it offers a command line interface that is almost identical to BWA and outputs SAM files (Sequence Alignment/Map format). CGAP-align is distributed under the GNU General Public License (GPL) with detailed documentation and source code freely available through Fudan University, BCM-HGSC and the Sourceforge web site. The pre-built indices for some public references (eg. Hg19) are also provided.

### 2.1 CGAP-align Evaluation

To evaluate the performance of CGAP-align, we have benchmarked it against BWA (version 0.5.9), SOAPv2 (version 2.20) and Bowtie2 (version 2.0.6), three of the most commonly used alignment programs. Other tools like Bowtie are not included because gapped alignments are currently not supported. All tools run on 4 threads. Both BWA and CGAP-align were evaluated on their ability to map 100 base pair long reads using either default settings or relatively loose settings that allow up to 5 edits and a gap extension of 3 base pairs (-n 5 -e 3 -l 25). The same data set is used for SOAPv2, allowing up to 5 mismatches and a gap size of 4 (-v 5 -g 4). This setting is looser than SOAPv2's default setting, which enables it to find more alignments. For bowtie2, default parameters are used (–sensitive) since no gap settings can be adjusted. In addition, two modes of CGAP-align, the first with STA alone (denoted CGAP-align) and the second with both DCDC and STA (denoted CGAP-align^*^), were evaluated. STA was built based on the human reference genome hg19 with 600 MB size. All of the experiments utilized the same AC automaton with a size of 1000 MB trained from independent WGS data for DCDC.

### 2.2 Evaluation on simulated data

The performance of BWA, CGAP-align, Bowtie2, and SOAPv2 on a simulated data set was examined. Wgsim, a program included in SAMtools [25], was used to generate 20 million simulated human genome shotgun 100 bp reads with a 2% error rate. As the exact position of each simulated read in the genome is known, both the sensitivity and specificity of the alignments could be precisely calculated.

As shown in Table 1, a significant speedup was achieved by CGAP-align while maintaining high mapping quality. We report both the absolute running time (Hours) and the percentage relative to BWA for all programs considered. When STA was used alone, CGAP-align was about 10% to 20% faster than BWA. Since the implementation of STA only accelerates the string matching step without affecting alignment, identical results were obtained between CGAP-align and BWA. Both programs produced mapping results with high accuracy and sensitivity. Sensitivity was measured by the true positive rate (TPR), which is defined as the overall percentage of correctly mapped reads relative to the number of input reads. In parallel, mapping specificity was measured by the positive predictive value (PPV), which indicates the percentage of correctly mapped reads relatively to the number of the reads reported as mapping correctly by the algorithm. Under default settings, 97.9% of the reads were accurately mapped with a PPV of 98.7%. The effect of STA was also evaluated under loose settings. A similar speed up was observed under this condition, and identical mapping results obtained by both CGAP-align and BWA as expected. For SOAPv2 and Bowtie, lower TPRs were observed than for CGAP-align although both run faster than CGAP-align.

**Table 1:** Evaluation on simulated data.

	Default Settings				Loose Settings			
Program	Hours	%	TPR (%)	PPV (%)	Hours	%	TPR (%)	PPV (%)
BWA	**2.08**	-	99.84	98.51	**3.70**	-	99.92	98.54
CGAP-align	1.77	85.10	99.84	98.51	3.29	88.92	99.92	98.54
CGAP-align^*^	1.80	86.54	99.84	98.51	2.89	78.11	99.92	98.54
Bowtie2	1.57	75.48	95.05	95.57	-	-	-	-
SOAPv2	0.95	45.67	84.95	98.21	-	-	-	-

The 10 million read pairs were mapped to the human genome. We recorded the run time (BWA in hours, CGAP-align and SOAP in percentage relative to BWA) on a 2.4 GHz Dual-Core AMD Opteron Processor 2216 HE with 4 threads running simultaneously (Hours & percentage relative to BWA), true positive rate (TPR) and positive predictive value (PPV). CGAP-align gave identical results to BWA with a shorter run time.

Surprisingly, despite the use of training data, little speedup observed after adding DCDC. As shown in Table 1, an almost identical speed was observed for CGAP-align^*^ under default settings. We found that in this case only 5% of the reads' D-arrays were improved by DCDC. This is because, in our simulated data set, most reads were aligned to the reference with only 1 or 2 edit operations, leaving little optimization space for DCDC. On the other hand, the DCDC itself consumed more time than the original D-array strategy, which led to a poorer overall performance. However, when we evaluated the programs on real data, where more mismatches and gaps exist, DCDC resulted in a significant speedup (Table 2).

**Table 2:** Evaluation on real data.

	Default Settings			Loose Settings		
Program	Hours	%	Conf (%)	Hours	%	Conf (%)
BWA-WES1	**3.350**	-	97.31	**7.130**	-	97.39
CGAP-align-WES1	2.887	86.18	97.31	6.375	89.41	97.39
CGAP-align^*^-WES1	2.698	80.54	97.31	3.813	53.48	97.33
Bowtie2-WES1	3.117	93.04	98.43	-	-	-
SOAPv2-WES1	1.076	32.12	93.85	-	-	-
BWA-WES2	**4.555**	**-**	96.84	**11.158**	-	96.92
CGAP-align-WES2	3.857	84.68	96.84	9.699	86.92	96.92
CGAP-align^*^-WES2	3.829	84.06	96.84	5.598	50.17	96.85
Bowtie2-WES2	3.700	81.23	98.02	-	-	-
SOAPv2-WES2	1.314	28.85	92.94	-	-	-
BWA-WGS1	**22.818**	**-**	91.84	**71.349**	-	92.26
CGAP-align-WGS1	20.817	91.23	91.84	66.262	92.87	92.26
CGAP-align^*^-WGS1	19.717	86.41	91.84	30.159	42.27	92.19
Bowtie2-WGS1	8.433	36.96	83.31	-	-	-
SOAPv2-WGS1	10.311	45.19	82.03	-	-	-
BWA-WGS2	**8.504**	**-**	93.69	**20.244**	-	93.82
CGAP-align-WGS2	7.629	89.71	93.69	19.218	94.93	93.82
CGAP-align^*^-WGS2	7.047	82.87	93.69	9.379	46.33	93.76
Bowtie2-WGS1	5.283	61.12	84.45	-	-	-
SOAPv2-WGS2	4.647	54.64	88.34	-	-	-

29.2 million read pairs (WES1), 31.6 million read pairs (WES2), 107.8 million read pairs (WGS1) and 86.3 million read pairs (WGS2) were mapped to the human genome. The run time (BWA in hours, CGAP-align and SOAP in percentage relative to BWA) on a 2.4 GHz Dual-Core AMD Opteron Processor 2216 HE with 4 threads running simultaneously (Hours & percentage relative to BWA), percent of confidently mapped reads including paired mapping (Conf) are shown.

Slightly different mapping results were obtained for CGAP-align^*^ compared with BWA under loose settings, due to the inconsistency of the cost metrics used during read alignment and D-array calculation. During the alignment of each read, BWA and CGAP-align consider the edit costs gap extensions as 0, as described in section 1.1.3. However, during the D-array calculation, these gap extension costs have to be counted as 1 because otherwise any read could be regarded as a single gap, and no value greater than 1 would be obtained in the D-array. As a result, pruning based on D-array misses some true positive alignments, a phenomenon that occurs in both BWA and CGAP-align. CGAP-align^*^, which computes a better D-array, omits a little more than BWA. In Discussion, we show a study case to illustrate such omissions. Fortunately, according to our results, in most cases CGAP-align^*^ produces the same alignment as BWA, and only infrequently omits marginal results. Such omissions are even more trivial as only the best candidates are presented in the final alignment. In this experiment, there were 8,544 reads with which BWA obtained an extra 33,105 hits in comparison to CGAP-align^*^. Of these reads, 8,306 of them (97.21%) contained multiple hits, and only 607 of them were correct.

When mapping to the human genome, SOAPv2 uses 5.4 GB of memory while BWA only uses about 3 GB. In our experiment, CGAP-align without DCDC consumed 3.6 GB of memory, and used 4.6 GB when DCDC was integrated. However, the memory consumption can be controlled by adjusting the index sizes for both STA and DCDC.

**Figure 4:**
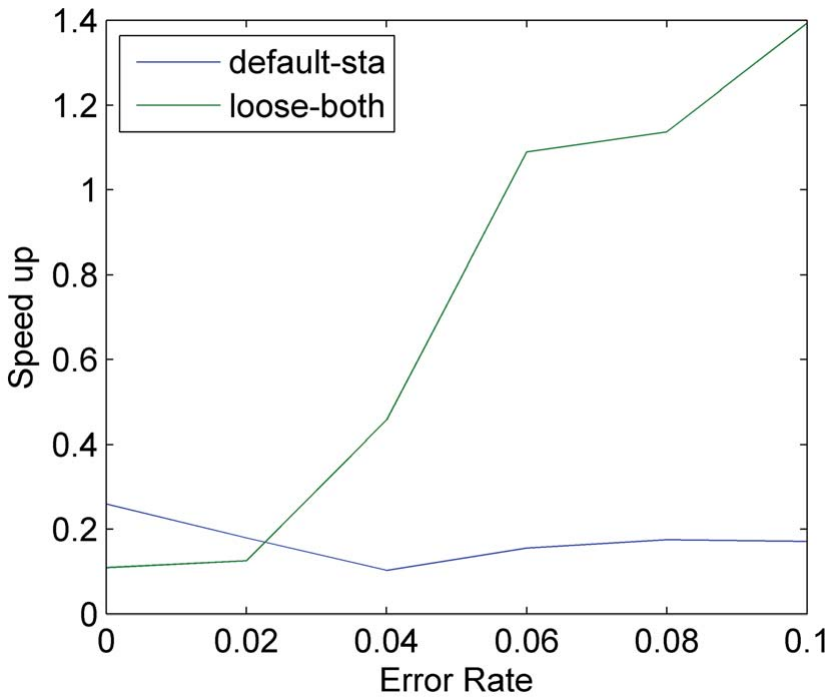
The error rates of the simulated reads vs. the speed up of CGAP-align over BWA, defined as ((T_BWA_-T_CGAP_)/T_CGAP_). Six sets of simulated human genome shotgun 100 bp reads with error rates from 0 to 0.1 were generated. The speed up when using STA under default settings and when using both STA and DCDC under loose settings is reported.

The relationship between the sequence error rate and the performance of STA and DCDC is shown in Figure 4. STA only optimizes the counting problem of the FM-index (i.e. determining the number of matches of a substring occurring in a reference) without modifying the locating problem (determining the positions in the reference where the matches occur). A maximal speed up of 26.0% was observed when no errors were introduced as almost all reads mapped precisely to their correct positions, reducing locating costs. As the error rates goes up, multiple hits may occur for a single read, which increases locating costs. The lowest speed up, 10.2%, was observed at an error rate of 0.04. With an error rate between 0.04 and 0.1, the performance of STA improved as the more and more reads became un-mappable, which again led to a decrease in locating costs. On the other hand, DCDC had poor performance with an error rate below 0.04, after which it rapidly improved as most of the missing alignments were avoided through pruning.

### 2.3 Evaluation on Real Data

We tested CGAP-align using whole exome sequence (WES) data from two individuals (29.2 million read pairs for WES1, 31.6 million read pairs for WES2) and whole genome sequence (WGS) data from a separate pair of individuals (107.8 million read pairs for WGS1 and 86.3 million read pairs for WGS2). All read pairs were produced by Illumina HiSeq with a 100 bp read length. Both the consumed time (Hours) and the frequency with which reads were confidently mapped (Conf) are reported in Table 2. For both CGAP-align and BWA, we use mapping quality threshold 10 to determine confident mappings. CGAP-align, utilizing STA alone, obtained a speed up of approximately 10% to 20% compared with BWA. Surprisingly, CGAP-align^*^, while achieving a small speed up with default settings, shortened the running time by more than 50% with loose settings, due to the high error rates of the reads in real data. While SOAPv2 was faster than all other candidates including CGAP-align^*^, it found less confidently mapped reads than BWA, especially for the WGS cases. On the WGS1 data, where a significant number of mismatches between the reads and reference were observed, CGAP-align and BWA confidently mapped almost 10% more reads than SOAPv2 did. In comparison to SOAPv2, CGAP-align^*^ further shortens the performance gap while maintaining a relatively high mapping rate. Overall, it possesses impressive mapping rates in WES while the performances when mapping WGS data still need to be improved.

## Discussion

Due to the enormous number of reads generated by the next generation sequencing technologies, the efficiency of read alignment has become a critical problem. In this article, we present CGAP-align, a variant of BWA, which doubles the speed of the alignment process. While SOAPv2 is also very fast, it is unable to map as many reads as CGAP-align. CGAP-align retains its high recall and precision even when the error rate of the reads is high. CGAP-align also outputs SAM files, allowing easy use of various downstream analysis tools.

CGAP-align requires a tunable amount of memory beyond that required by BWA, which equals to the size of the additional indices for STA and DCDC. To make DCDC work, a set of training reads are needed for CGAP-align to build the DCDC index. In practice, any FASTA file containing reads that are supposed to be mapped to the reference is qualified to be the training dataset for that reference. However, we recommend not using FASTA files larger than 2 GB to reduce memory cost and time of DCDC index construction.

It is very easy to migrate from using BWA to CGAP-align. For each reference sequence, only a single command line is needed to build the additional indices used by CGAP-align. After that, CGAP-align has an identical interface to that of BWA. In addition, CGAP-align is backwards compatible with BWA, so BWA can do read alignment using the FM-index produced by CGAP-align.

**Figure 5:**
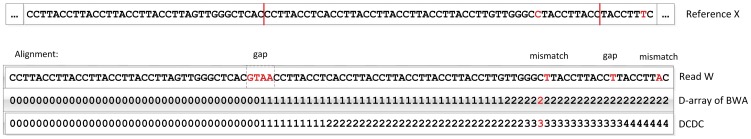
A case study for the inconsistency of the alignment results with loose setting (5 edit costs at most) where BWA successfully finds an alignment for the read *W* while CGAP-align* fails. The red color on the first line indicates the edit operations in the alignment found by BWA. In this particular alignment, we have 2 gaps and 2 mismatches. According to the cost metrics used during the alignment stage where the cost of gap extension is considered as 0, we need 4 edit costs to map *W* to *X*, satisfying the loose setting. The D-arrays calculated by BWA and DCDC are listed below. Both of them are strictly correct under D-array's cost metrics where the cost of a gap extension is 1.

The main concern with using CGAP-align^*^ is that it occasionally misses alignments called by BWA. Figure 5 further investigates a real example of the cause of these differences. Consider CGAP-align^*^ mapping during the alignment depicted in Figure 5 under loose settings, where at most 5 edit costs are allowed. The backward search starts from right to left. When the search goes to the second mismatch, 3 edit costs have been counted. The D-array element at the next position (red number on the forth row) also has a value of 3, indicating that at least 6 edit costs are needed to map *W* to *X* according to DCDC's D-array. As a result, this alignment is pruned by CGAP-align^*^. However, at the same position in BWA's case, the D-array value is 2 (telling us that at least 5 edit costs are needed), which does not trigger the pruning, leading to the discrepancy between the alignments. From the mechanism behind this example, we can see that such discrepancies are quite random and partially depend on the gap positions in the alignment. As previously noted, only a very small fraction of reads encounter this problem and the pruned alignments are typically quite marginal.
